# Prevalence and correlates of frailty among older adults: findings from the German health interview and examination survey

**DOI:** 10.1186/s12877-015-0022-3

**Published:** 2015-03-08

**Authors:** Amanda K Buttery, Markus A Busch, Beate Gaertner, Christa Scheidt-Nave, Judith Fuchs

**Affiliations:** Department of Epidemiology and Health Monitoring, Robert Koch Institute, General-Pape-Str. 64, Berlin, 12101 Germany

**Keywords:** Frailty, Aging, Elderly, Prevalence, Community-dwelling, Germany

## Abstract

**Background:**

Despite having the third highest proportion of people aged 60 years and older in the world, Germany has been recently reported as having the lowest prevalence of frailty of 15 European countries. The objective of the study is to describe the prevalence of frailty in a large nationwide population-based sample and examine associations with sociodemographic, social support and health characteristics.

**Methods:**

We performed a **c**ross-sectional analysis of the first wave of the German Health Interview and Examination Survey for Adults (DEGS1) conducted 2008–2011. Participants were 1843 community-dwelling people aged 65–79 years. Frailty and pre-frailty were defined, according to modified Fried criteria, as 3 and more or 1–2 respectively, of the following: exhaustion, low weight, low physical activity, low walking speed and low grip strength. The Oslo-3 item Social Support Scale (OSS-3) was used. Patient Health Questionnaire (PHQ-9) measured depressive symptoms and the Digit Symbol Substitution Test (DSST) measured cognition. Associations between participants’ characteristics and frailty status were examined using unadjusted and adjusted multinomial logistic regression models estimating relative risk ratios (RRR) of frailty and pre-frailty.

**Results:**

The prevalence of frailty among women was 2.8% (CI 1.8-4.3) and pre-frailty 40.4% (CI 36.3-44.7) and among men was 2.3% (CI 1.3-4.1) and 36.9% (CI 32.7-41.3) respectively. Independent determinants of frailty, from unadjusted models, included older age, low socioeconomic status, poor social support, lower cognitive function and a history of falls. In adjusted models current depressive symptoms (RRR 12.86, CI 4.47-37.03), polypharmacy (RRR 7.78, CI 2.92-20.72) and poor hearing (RRR 5.38, CI 2.17-13.35) were statistically significantly associated with frailty.

**Conclusions:**

Frailty prevalence is relatively low among community-dwelling older adults in Germany. Modifiable characteristics like low physical activity provide relevant targets for individual and population-level frailty detection and intervention strategies.

## Background

The complex condition of frailty continues to gain increased attention in the search to improve healthy life expectancies for aging populations and healthcare for older people. Frailty develops as a result of age-related decline in various physiological systems leading to a state of vulnerability and impaired ability to adapt to external stressors and an increased risk of dependency and adverse outcomes [1]. Recent findings from the Survey of Health, Aging and Retirement in Europe (SHARE) wave 4 (2010–11) report that Germany, a country with the third highest proportion of people aged 60 years and older in the world [2], has the lowest prevalence of frailty among 15 European countries [3]. Prevalence estimates for frailty for people aged 50 years and older were less than 1% for women and men in Germany and were calculated within the context of activity limitation using the SHARE-Frailty Index (FI) [3]. For those aged 70 years, life expectancy estimates with frailty were very low in Germany with men estimated as averaging 0.1 years (0.7% of life expectancy) and women 0.4 years (2.3% of life expectancy) with frailty [3]. Further exploration of frailty and its correlates in Germany is needed to improve our understanding of intrinsic and extrinsic factors associated with frailty and resilience that maintains individual well-being and independence [4].

Characterising population subgroups at risk of frailty and relationships between frailty, social circumstances, depressive symptoms, cognition and other health-related factors will help identify modifiable intervention targets which may reduce the burden of frailty for individuals and help give directions for public health policy [5]. Using national health survey data we investigate sociodemographic and health determinants of frailty in a population-based sample of community-dwelling older adults in Germany.

## Methods

The German Health Interview and Examination Survey for Adults (DEGS) survey is part of the continuous national health monitoring system carried out by the Robert Koch Institute and provides representative data on the health of adults in Germany at regular intervals [6]. The first wave (DEGS1) was conducted from November 2008 to December 2011 and the design, objectives and methods have been described in detail elsewhere [6]. Briefly, DEGS1 has a mixed design permitting both cross-sectional and longitudinal analyses. Persons aged 18–79 years randomly selected from local population registries and former German National Health Interview and Examination Survey participants in 1998 (GNHIES98) [6] were invited to participate. In DEGS1, 8152 non-institutionalised adults participated including 4193 first-time participants aged 18–79 years (response rate 42%) and 3959 previous participants of GNHIES98 aged 28–91 years (response rate 62%) [6].

DEGS1 included: standardised physician-administered computer-assisted personal interview (CAPI) with details on pre-existing physician-diagnosed health conditions; self-administered questionnaires; a range of physical, laboratory and other measurements; and robust data collection of medicines use. Complete interview and examination data for cross-sectional analyses were initially available for 7116 participants aged 18–79 years [6]. One participant was later excluded from all further analyses following withdrawal of informed consent. For this study we focused on older adults, those aged 65–79 years with interview and examination data (n = 1853). Participants whose frailty status could not be determined due to missing data on three or more of the five items used to measure frailty [7] were excluded (n = 10).Therefore, the final study sample was 1843 participants.

Frailty was defined and measured based on modified criteria used by Fried and colleagues in the Cardiovascular Health Study [7]; the most frequently used method for quantifying frailty in population-based studies [8]. Participants with three or more of the following characteristics: self-reported exhaustion, low weight, low physical activity, low walking speed and low grip strength were classified as frail [7]. Participants fulfilling one or two of the criteria were classified as ‘pre-frail’ and ‘non-frail’ if they fulfilled none. Similar to others [5,9], the measurement tools used to characterise the individual components of frailty were slightly adapted due to data availability and were determined as follows:

### Self-reported exhaustion

Exhaustion was measured using a single item from the validated German language version of the Medical Outcome Short Form-36 (SF-36) [10]. Participants were asked: ‘How much of the time during the past four weeks did you have a lot of energy?’ Those responding ‘none’ or ‘little of the time’ were classified as having exhaustion consistent with other studies [11,12].

### Low weight

No question related to unintentional weight loss was available in DEGS1. We used Body Mass Index (BMI) as an alternative consistent with others [9,13,14]. Those with a BMI of less than 23 were considered low weight akin to European studies investigating frailty [9,13] and taking into account contemporary obesity prevalence among older adults in Germany.

### Low physical activity

Standardised questions related to physical activity and sports were included in DEGS1 [15]. Participants reporting performing no sports in the previous three months and no physical activity on any day of the week requiring the person to start to sweat or get out of breath were classified as having low physical activity.

### Low walking speed

The timed up and go test is a reliable and valid test with a standardised protocol for quantifying functional mobility [16]. Participants taking 15 seconds or more were classified as having a low walking speed. This cut point has demonstrated 100% specificity for identifying all pre-frail or frail individuals according to Fried’s criteria in a large cohort of older people [17].

### Low grip strength

Isometric grip strength was measured using a hand-held dynamometer (Smedley, Scandidact, Denmark, 100 kg). Two values were recorded for each hand. Maximum grip strength from all attempts was used for analyses. Low grip strength was determined using sex and BMI specific cut points specified by Fried and colleagues [7]. Few participants had valid grip strength measures but missing BMI values (n = 15). For these participants, low grip strength was determined using established grip strength values for sarcopenia (<20 kg for women and <30 kg for men) consistent with others [5].

Socio-demographic variables included age, sex and living alone. Socioeconomic status (SES) was classified as low, middle and high using an established index including information on education, professional status and household income [6]. Self-perceived levels of social support were assessed using the Oslo-3 Social Support Scale (OSS-3) with poor social support defined as <9 points (range 3–14) [18]. Additionally, participants were asked: ‘Do you think you need more help in your daily routine than you currently receive?’ Those responding ‘yes’ were classified as having self-reported lack of help.

Current depressive symptoms within the last 2 weeks were assessed using the 9-item depression module of the German version of the Patient Health Questionnaire (PHQ-9) with those scoring 10 or more points (range 0–27) as having current depressive symptoms [19]. Cognitive function was assessed using the paper-and-pencil Digit Symbol Substitution Test (DSST) from the German version of the Wechsler Adult Intelligence Scale, 3rd revision (WAIS-III) [20]. Participants are provided with a key showing unique graphic symbols corresponding to numerical digits and copy symbols onto a scoring sheet within 120 seconds (score range 0–133).

Self-rated health was measured using a standard item from the SF-36 [10] and dichotomised as excellent/very good/good versus fair/poor. Self-reported chronic diseases (“Has a doctor ever diagnosed you as having..?”) included: coronary heart disease (CHD) (including myocardial infarction (MI), angina or other CHD), stroke, heart failure, chronic renal disease, cancer, osteoarthritis, rheumatoid arthritis and osteoporosis. Hypertension and diabetes were determined based on self-reported history of disease and intake of antihypertensive [21] or antidiabetic medication [22]. Polypharmacy was defined as the use of 5 and more prescription medicines in the 7 days prior to examination [23]. History of falls was defined as those reporting two or more falls in the past 12 months. Self-reported poor hearing and poor vision were recorded. Health risk behaviours included current smoking status and alcohol consumption (first item from Alcohol Use Disorders Identification Test-Consumption [24]).

### Statistical analyses

To describe the sample and the prevalence of frailty participants’ characteristics were stratified by sex. To describe the sample according to their frailty status, proportions, means, and respective 95% confidence intervals (CI) are presented. To determine differences between (a) non-frail versus pre-frail and (b) non-frail versus frail groups, separate unadjusted multinomial regression analyses were conducted using the non-frail group as the reference group. Then, all multinomial regression models were adjusted for age (in years), sex and SES. Both unadjusted and adjusted models, including relative risk ratios (RRR) and 95% CIs are presented. Participants with missing values were not included in analyses.

In order to report population estimates, all analyses were conducted with a weighting factor which corrects deviations in the sample from the population structure (as of 31 Dec 2010) with regard to age, sex, region and nationality, as well as community type and education. Calculation of the weighting factor also considered re-participation probability of GNHIES98 participants, based on a logistic regression model. To take into account the weighting as well as the correlation of the participants within a community, the confidence intervals were determined with complex samples procedures in SPSS-20 or survey procedures in STATA 12.1.

### Ethics

DEGS1 was approved by the Charité-Universitaetsmedizin Berlin ethics committee (No. EA2/047/08) and conducted according to guidelines provided by the Federal and State Commissioners for Data Protection. Participants provided written informed consent prior to interview and examination.

## Results

Table 1 presents summary characteristics of study participants. Table 2 presents the prevalence of frailty and its components by sex: 2.3% of men and 2.8% of women were frail, 36.9% of men and 40.4% of women were prefrail. Frailty status did not differ statistically significantly between women and men. Low physical activity (men 24.1%, women 21.1%) and low grip strength (men: 10.8%, women: 12.6%) were the most common frailty components for both women and men. With regard to frailty components, women were significantly more likely to have low weight.

**Table 1:** **Characteristics of German Health and Examination Survey 2008–2011 (DEGS1) participants aged 65–79 years included in this study**

	**Men (n = 919)**	**Women (n = 924)**	**Total (n = 1843)**
Age groups in years (%, CI)			
65-69	36.3 (32.6-40.1)	33.6 (30.2-37.3)	34.8 (32.4-37.4)
70-74	43.2 (39.2-47.2)	42.9 (39.0-46.9)	43.0 (40.1-46.0)
75-79	20.6 (17.5-24.0)	23.4 (20.0-27.2)	22.1 (19.6-24.9)
Socioeconomic status (SES) (%, CI)			
low	21.7 (17.9-26.2)	29.1 (24.7-33.9)	25.7 (22.3-29.4)
medium	57.3 (52.8-61.7)	61.7 (57.2-66.0)	59.7 (56.1-63.1)
high	21.0 (17.7-24.7)	9.2 (7.2-11.6)	14.6 (12.6-17.0)
Living alone (%, CI)	9.6 (7.3-12.6)	34.0 (29.8-38.4)	22.8 (20.1-25.7)
Poor social support (%, CI)	16.8 (13.3-21.0)	16.2 (13.1-19.9)	16.5 (14.1-19.2)
Self-reported lack of help (%, CI)	2.0 (1.3-3.1)	8.2 (6.2-10.8)	5.3 (4.2-6.8)
Current depressive symptoms (%, CI)	4.2 (2.8-6.2)	7.3 (5.2-10.2)	5.9 (4.5-7.6)
Digit Symbol Substitution Test (mean score; CI)	42.6 (41.3-43.9)	45.0 (43.6-46.5)	43.9 (42.9-44.9)
Self-rated health (fair/poor) (%, CI)	27.4 (23.4-31.8)	28.9 (25.1-33.2)	28.2 (25.4-31.3)
**Chronic diseases** (%, CI)			
coronary heart disease	27.6 (23.7-31.8)	14.5 (11.9-17.6)	20.6 (18.2-23.2)
stroke	7.2 (5.1-10.0)	6.0 (4.2-8.4)	6.5 (5.1-8.4)
heart failure	13.2 (10.2-16.9)	12.3 (9.5-15.9)	12.7 (10.5-15.4)
hypertension	70.3 (65.9-74.4)	71.6 (68.1-74.9)	71.0 (68.2-73.7)
diabetes	21.0 (17.5-25.0)	18.6 (15.4-22.2)	19.7 (17.3-22.3)
chronic renal disease	3.2 (2.2-4.8)	5.0 (3.3-7.5)	4.2 (3.0-5.7)
cancer	15.8 (13.2-18.9)	14.6 (11.9-17.8)	15.2 (13.2-17.4)
osteoarthritis	32.1 (28.3-36.2)	51.6 (47.7-55.4)	42.6 (39.9-45.3)
rheumatoid arthritis	5.0 (3.0-8.2)	4.3 (3.0-6.0)	4.6 (3.4-6.1)
osteoporosis	3.5 (2.4-5.3)	23.2 (19.4-27.5)	14.1 (12.0-16.5)
Mean number of chronic diseases (CI)	2.0 (1.8-2.1)	2.2 (2.1-2.3)	(2.1 2.0-2.2)
Number of chronic diseases (%, CI)			
none	12.5 (9.7-15.9)	6.8 (5.1-8.9)	9.4 (7.7-11.4)
1	28.0 (24.4-31.9)	27.1 (23.4-31.2)	27.5 (24.9-30.2)
2	29.8 (25.9-34.0)	30.6 (26.8-34.8)	30.2 (27.5-33.1)
3 or more	29.7 (25.7-34.0)	35.5 (31.5-39.7)	32.8 (29.8-36.1)
Polypharmacy (≥5 prescribed medicines) (%)	42.1 (37.8-46.5)	50.5 (46.6-54.3)	46.6 (43.7-49.5)
Falls (≥2) in past 12 months (%, CI)	6.1 (4.3-8.5)	10.5 (8.2-13.4)	8.5 (6.9-10.3)
Poor hearing (%, CI)	1.2 (0.6-2.2)	6.1 (4.2-8.7)	3.8 (2.8-5.3)
Poor vision (%, CI)	7.4 (5.5-9.8)	5.6 (4.0-7.8)	6.4 (5.0-8.1)
**Health risk behaviours** (%, CI)			
body mass index (BMI), kg/m^2^			
underweight (BMI < 18.5)	0.1 (0.0-0.4)	1.3 (0.4-4.3)	0.7 (0.2-2.3)
normal (BMI 18.5 < 25)	17.0 (14.2-20.2)	22.2 (19.2-25.4)	19.7 (17.7-21.9)
overweight (BMI 25 to <30)	51.4 (46.9-55.8)	37.7 (33.9-41.8)	44.1 (41.2-46.9)
obese (BMI ≥ 30)	31.6 (27.2-36.4)	38.9 (35.0-42.9)	35.5 (32.4-38.7)
currently smoking	10.9 (8.6-13.7)	8.6 (6.5-11.3)	9.7 (8.1-11.4)
current alcohol consumption	91.4 (88.6-93.6)	77.5 (73.2-81.3)	84.0 (81.2-86.5)

Figures are weighted population estimates expressed in per cent unless otherwise indicated.

CI: 95% confidence interval.

Not all percentages add to 100 due to rounding error.

**Table 2:** **Prevalence of frailty and pre-frailty and its components**

	**Men (n = 919)% (CI)**	**Women (n = 924)% (CI)**	**Total (n = 1843)% (CI)**
Frailty status			
frail	2.3 (1.3-4.1)	2.8 (1.8-4.3)	2.6 (1.8-3.6)
pre-frail	36.9 (32.7-41.3)	40.4 (36.3-44.7)	38.8 (35.9-41.8)
non-frail	60.8 (56.3-65.0)	56.7 (52.6-60.8)	58.6 (55.6-61.5)
Frailty components			
self-reported exhaustion	5.5 (3.7-8.1)	9.7 (7.5-12.5)	7.8 (6.2-9.6)
low weight^a^	4.7 (3.1-7.0)	10.3 (8.0-13.2)	7.7 (6.2-9.5)
low physical activity	24.1 (20.2-28.4)	21.1 (17.7-25.0)	22.5 (19.8-25.5)
low walking speed^b^	6.9 (4.9-9.5)	8.9 (6.5-12.0)	8.0 (6.3-10.0)
low grip strength	10.8 (8.3-13.9)	12.6 (9.7-16.2)	11.8 (9.6-14.3)

Figures are weighted population estimates expressed in per cent unless otherwise indicated.

CI: 95% confidence interval.

Not all percentages add to 100 due to rounding error.

^a^Body mass index < 23.

^b^Timed up and go test 15 seconds or more.

Table 3 shows the results of the unadjusted and adjusted multinomial regression models of associations between participants’ characteristics and frailty status. Significant associations were found for all variables except sex and living alone. Associations were consistent across unadjusted and adjusted models. Frail participants were older, were more likely to have low SES and poor social support, more often reported they lacked help, more often had current depressive symptoms and had lower mean DSST scores compared to non-frail participants. In addition, frail participants reported worse self-reported health, more chronic diseases, polypharmacy, more falls in the past 12 months, poor hearing, poor vision and reported not consuming alcohol compared to non-frail participants. The directional associations between non-frail and pre-frail participants were similar for nearly all variables as for non-frail and frail participants, although RRRs were lower. However, there were two exceptions: (a) there was no significant association between non-frail and pre-frail groups for poor hearing and (b) pre-frail participants reported more frequently currently smoking compared to non-frail and frail participants.

**Table 3:** **Prevalence and associations between participants’ characteristics and frailty status in unadjusted and adjusted multinomial regression models**

				**Unadjusted model**		**Adjusted model** ^**a**^	
				**Not frail versus**		**Not frail versus**	
	**Non-frail**	**Pre-frail**	**Frail**	**Pre-frail**	**Frail**	**Pre-frail**	**Frail**
	**n = 1139**	**n = 659**	**n = 45**				
	**%**	**%**	**%**	**RRR (CI)**	**RRR (CI)**	**RRR (CI)**	**RRR (CI)**
Age groups in years							
65-69	36.8	33.1	17.9	Ref.	Ref.	Ref.	Ref.
70-74	45.0	40.3	40.3	0.99 (0.77-1.29)	1.85 (0.69-4.91)	0.94 (0.72-1.22)^b^	1.64 (0.64-4.18)^b^
75-79	18.3	26.7	41.8	1.62 (1.15-2.29)	4.72 (1.79-12.41)	1.49 (1.04-2.13)^b^	4.03 (1.63-9.98)^b^
Sex							
men	47.9	43.9	41.6	Ref.	Ref.	Ref.	Ref.
women	52.1	56.1	58.4	1.17 (0.90-1.52)	1.29 (0.61-2.76)	1.08 (0.82-1.41)^c^	1.05 (0.50-2.19)^c^
Socioeconomic status (SES)							
low	19.9	32.4	56.7	Ref.	Ref.	Ref.	Ref.
medium	62.4	57.2	35.3	0.56 (0.41-0.78)	0.20 (0.10-0.40)	0.57 (0.41-0.79)^d^	0.21 (0.10-0.42)^d^
high	17.7	10.4	8.0	0.36 (0.24-0.54)	0.16 (0.04-0.66)	0.38 (0.25-0.58)^d^	0.19 (0.05-0.75)^d^
Living alone							
no	78.2	76.2	69.8	Ref.	Ref.	Ref.	Ref.
yes	21.8	23.8	30.2	1.12 (0.84-1.49)	1.55 (0.73-3.31)	1.02 (0.76-1.37)	1.31 (0.57-3.01)
Poor social support							
no	88.3	78.1	56.0	Ref.	Ref.	Ref.	Ref.
yes	11.7	21.9	44.0	2.12 (1.43-3.15)	5.94 (2.75-12.83)	1.99 (1.34-2.95)	4.96 (2.21-11.12)
Self-reported lack of help							
no	97.6	91.6	76.1	Ref.	Ref.	Ref.	Ref.
yes	2.4	8.4	23.9	3.67 (2.11-6.38)	12.51 (4.97-31.45)	3.50 (1.96-6.25)	12.18 (4.73-31.35)
Current depressive symptoms							
no	97.2	90.7	73.5	Ref.	Ref.	Ref.	Ref.
yes	2.8	9.3	26.5	3.55 (2.04-6.16)	12.47 (5.09-30.56)	3.64 (2.03-6.53)	12.86 (4.47-37.03)
Mean Digit Symbol Substitution Test	46.0	41.4	32.6	0.98 (0.97-0.98)	0.93 (0.90-0.95)	0.98 (0.97-0.99)^e^	0.94 (0.91-0.97)^f^
Self-reported health (fair/ poor)							
no	79.3	63.5	22.6	Ref.	Ref.	Ref.	Ref.
yes	20.7	36.5	77.4	2.20 (1.67-2.92)	13.16 (5.21-33.20)	2.21 (1.66-2.94)	13.30 (5.15-34.31)
Chronic diseases^g^							
mean	1.9	2.3	3.3	1.24 (1.13-1.36)	1.94 (1.57-2.40)	1.23 (1.11-1.35)	1.92 (1.56-2.35)
3 or more diseases							
no	73.2	60.0	39.7	Ref.	Ref.	Ref.	Ref.
yes	26.9	40.0	60.3	1.82 (1.42-2.33)	4.14 (1.91-8.97)	1.76 (1.63-2.26)	3.79 (1.72-8.31)
Polypharmacy							
no	58.5	48.3	14.2	Ref.	Ref.	Ref.	Ref.
yes	41.5	51.7	85.8	1.51 (1.20-1.89)	8.54 (3.35-21.77)	1.47 (1.17-1.86)	7.78 (2.92-20.72)
Falls (≥2) in past 12 months							
no	93.8	88.9	79.2	Ref.	Ref.	Ref.	Ref.
yes	6.2	11.1	20.8	1.89 (1.16-3.08)	3.97 (1.53-10.26)	1.84 (1.15-2.96)	4.31 (1.65-11.26)
Poor hearing							
no	94.4	93.6	74.9	Ref.	Ref.	Ref.	Ref.
yes	5.6	6.4	25.1	1.15 (0.71-1.87)	5.69 (2.27-14.23)	1.14 (0.71-1.84)	5.38 (2.17-13.35)
Poor vision							
no	97.7	91.4	88.7	Ref.	Ref.	Ref.	Ref.
yes	2.3	5.6	11.3	2.50 (1.30-4.79)	5.31 (1.53-18.42)	2.24 (1.20-4.14)	3.81 (1.09-13.34)
Currently smoking							
no	91.9	87.9	91.5	Ref.	Ref.	Ref.	Ref.
yes	8.1	12.1	8.5	1.56 (1.02-2.38)	1.06 (0.25-4.42)	1.65 (1.08-2.50)	1.32 (0.30-5.81)
Current alcohol consumption							
no	12.7	20.6	38.2	Ref.	Ref.	Ref.	Ref.
yes	87.3	79.4	61.8	0.56 (0.41-0.77)	0.24 (0.11-0.51)	0.61 (0.44-0.85)	0.30 (0.11-0.82)

Figures are weighted population estimates expressed in per cent unless otherwise indicated. Not all percentages add to 100 due to rounding error.

RRR: relative risk ratio.

CI: 95% confidence interval.

Ref.: reference category.

^a^Multinomial logistic regression models for each variable adjusted for age, sex and low socio economic status (SES) unless otherwise stated, ^b^adjusted for sex and SES only, ^c^adjusted for age and SES only, ^d^adjusted for age and sex only, ^e^alternatively adjusted for age, sex, education (not SES) RRR = 0.98, CI: 0.97-0.99, ^f^alternatively adjusted for age, sex, education (not SES) RRR = 0.92, CI: 0.90-0.95, ^g^Ten conditions included coronary heart disease, stroke, heart failure, hypertension, diabetes, chronic renal disease, cancer, osteoarthritis, rheumatoid arthritis and osteoporosis.

## Discussion

Our prevalence estimates of frailty (2.6%) and pre-frailty (38.8%) are lower than most German, European and international studies, using Fried criteria to define frailty. For example, findings from the SHARE cohort (wave 1 in 2004) estimated 12.1% of people aged 65 years as frail in Germany [25]. In the SHARE study, frailty was measured using the SHARE-FI, an instrument based on Fried criteria [7] but using different components e.g. walking speed was measured by self-reported items related to mobility limitations and not objective measurement such as gait speed or the timed up and go.

DEGS1 included only participants aged 65 to 79 years and no home visits were offered. This might have resulted in the low estimates of frailty. In contrast, the SHARE study has no upper age limit for participant inclusion and incorporates data collection from home visits. These differences in study design, participant inclusion criteria and heterogeneity of instruments used to operationalise frailty [5,26] highlight that careful attention is required to make meaningful comparisons between frailty prevalence studies. However, our findings are comparable the Lausanne Cohort (Lc65+) longitudinal study using similar methods of data collection at a study centre and reporting 2.5% of participants as frail and 26.5% as pre-frailty [27], albeit in a slightly younger sample (age range 65–70 years) than our study. In a systematic review comparing and pooling international frailty prevalence data, although estimates varied widely, the overall weighted average prevalence of frailty was 9.9% (range 4.0%-17.0%) in community-dwelling adults aged 65 years and over using definitions of frailty based on Fried criteria [26]. Therefore, we cautiously interpret our prevalence estimates as relatively low in an international context.

We found no statistically significant associations between sex and frailty contrasting with numerous studies reporting higher frailty prevalence among women [26]. Our non-significant findings are likely a result of the low prevalence of frailty in our study and may represent Type II error. Women demonstrated higher prevalence of all frailty components except low physical activity. The frailty phenotype is intrinsically connected with muscle strength measurement, both in terms of grip strength and walking speed, and sex differences in sarcopenia and its causal factors [28] may underpin sex differences in frailty prevalence. Alternatively, our non-significant findings between sex and frailty may be explained by women performing higher levels of physical activity (as they reported), thereby buffering the relative sex differences in sarcopenia between ages 65–79 years and delaying the age of onset of frailty to later life i.e. beyond the upper age limit of our study.

We found an association between SES and frailty indicating a social gradient in frailty, which may be mediated by conditions that occur more frequently among disadvantaged individuals [3,5]. Although previous studies have reported associations between living alone and frailty [9], we found no such association. However, we found strong associations between low levels of social support and self-reported lack of help and frailty highlighting that these factors may contribute more to frailty status than living alone in Germany.

Consistent with previous research we found significant relationships between current depressive symptoms [9,11,29] and lower cognition [9,29,30] and frailty. Unsurprisingly, we found a clear incremental association between the number of chronic diseases, pre-frailty and frailty. Polypharmacy showed a stronger relationship to frailty in terms of relative risk than having more chronic diseases therefore it may be a relevant modifiable clinical target to reduce frailty. Poor hearing was strongly associated with frailty, but not with pre-frailty status in our study. We found smoking status was particularly associated with pre-frailty and lower alcohol consumption was associated with being frail consistent with others [5,9].

Low physical activity and low grip strength were the two most common components of frailty signposting that methods to improve physical activity and muscle strength need greater attention in research examining the frailty burden at individual and population levels. Other relevant targets for specific frailty detection and intervention studies relate to socioeconomic status, social support, depressive symptoms, cognition, falls, polypharmacy and poor hearing.

Our study has a number of strengths and limitations. The standardised methods used in DEGS1 ensure high quality data collection in a representative sample of the German population. However, the survey focus is on the community-dwelling population; excluding residents of institutions and those unable to visit the study centres who are more likely to be frail and therefore our findings may under-estimate frailty at the population level. We used Fried and colleagues’ criteria to classify frailty; however, we made minor modifications to the original components due to data availability. We recognise complex relationships exist between BMI, obesity and frailty status and our methods of using BMI as a proxy for unintentional weight loss, although consistent with numerous others [9,13,14] may have resulted in misclassification of some older adults with obesity as not frail. Our results should be interpreted with caution as the absolute numbers of participants with frailty was low and restricted analyses. For example, we were unable to undertake more detailed sex-specific analyses. However, recognising the importance of age, sex and SES status from previous frailty studies [5,7,9] we performed restricted multinomial regression models adjusting for these key factors. This study considered only cross-sectional relationships between participants’ characteristics and frailty. Temporal relationships between frailty onset and outcomes were not examined.

## Conclusions

The community-dwelling German population aged 65–79 years demonstrates intriguingly low levels of frailty at the end of the 2010’s and warrants further examination with epidemiological studies using consistent methods to facilitate comparisons of data and data pooling. We found significant associations between participants’ characteristics and levels of frailty. Modifiable characteristics might provide clinically relevant targets for interventions; particularly in the case of poor social support, current depressive symptoms, cognition, falls prevention, polypharmacy and poor hearing.

## Abbreviations

DEGS1: German Health Interview and Examination Survey for Adults
GNHIES98: German National Health Interview and Examination Survey participants in 1998
SHARE: Survey of Health, Aging and Retirement in Europe
OSS-3: Oslo-3 item Social Support Scale
PHQ-9: Patient Health Questionnaire
DSST: Digit Symbol Substitution Test
RRR: Relative risk ratios
CI: 95% confidence interval
SF-36: Medical Outcome Short Form-36
BMI: Body Mass Index
SES: Socioeconomic status
CHD: Coronary heart disease
